# Immune Response to Vaccination against COVID-19 in Breastfeeding Health Workers

**DOI:** 10.3390/vaccines9060663

**Published:** 2021-06-17

**Authors:** Katarzyna Jakuszko, Katarzyna Kościelska-Kasprzak, Marcelina Żabińska, Dorota Bartoszek, Paweł Poznański, Dagna Rukasz, Renata Kłak, Barbara Królak-Olejnik, Magdalena Krajewska

**Affiliations:** 1 Department of Nephrology and Transplantation Medicine, Wroclaw Medical University, 50-367 Wroclaw, Poland; katarzyna.jakuszko@umed.wroc.pl (K.J.); katarzyna.koscielska-kasprzak@umed.wroc.pl (K.K.-K.); marcelina.zabinska@umed.wroc.pl (M.Ż.); dorota.bartoszek@umed.wroc.pl (D.B.); dagna.rukasz@umed.wroc.pl (D.R.); renataklak@wp.pl (R.K.); magdalena.krajewska@umed.wroc.pl (M.K.); 2 Department of Neonatology, Wroclaw Medical University, 50-367 Wroclaw, Poland; barbara.krolak-olejnik@umed.wroc.pl

**Keywords:** breast milk, breastfeeding, anti-SARS-CoV-2 antibodies, lactation, COVID-19, vaccination, mRNA vaccine

## Abstract

Background: Initially, there were no data on the safety of COVID-19 vaccines in lactating women. The aim of our study was to evaluate the immune response to COVID-19 vaccinations in breastfeeding women. Methods: The study included 32 breastfeeding women who, regardless of the study, had decided to be vaccinated. Maternal serum and breast milk samples were simultaneously collected on days 8 ± 1, 22 ± 2, 29 ± 3, and 43 ± 4 after the first dose of the vaccine. The immune response was assessed by determining the presence of anti-SARS-CoV-2 IgG and IgA. Results: The breast milk IgG level was detectable (6.50 ± 6.74, median 4.7, and maximum 34.2 BAU/mL) and highly correlated to serum IgG level (r_S_ 0.89; *p* < 0.001). The breast milk ratio of IgA to the cut-off value was higher in serum IgA-positive (4.18 ± 3.26, median 2.8, and maximum >10) than in serum IgA-negative women (0.56 ± 0.37, median 0.5, and maximum 1.6; *p* < 0.001). The highest concentrations of serum and breast milk antibodies were observed on day 29 ± 3 with a decrease on day 43 ± 4. Conclusion: The immune response to the vaccination against SARS-CoV-2 is strongest 7 ± 3 days after the second dose of the vaccine. Lactating mothers breastfeeding their children after vaccination against SARS-CoV-2 may transfer antibodies to their infant.

## 1. Introduction

The coronavirus disease 2019 (COVID-19) vaccine is one of the key elements of the strategy to hold off the pandemic and the further spread of the severe acute respiratory syndrome coronavirus 2 (SARS-CoV-2). The vaccine triggers the natural production of antibodies in the human body, and also stimulates the immune cells to protect against COVID-19.

According to the recommendations of the Advisory Committee on Immunization Practices (ACIP), vaccinations against COVID-19 were carried out initially in healthcare workers and residents of long-term care facilities [1]. An mRNA vaccine against COVID-19 (with modified nucleosides) is a single-stranded messenger RNA (mRNA), produced by in vitro cell-free transcription on a DNA template, encoding the SARS-CoV-2 spike protein (S).

As with any vaccine, the contraindication to administer an mRNA vaccine is an allergic reaction to a previous dose or an allergy to any of its ingredients (e.g., polyethylene glycol 2000). Vaccination should be postponed until clinical symptoms resolve in subjects presenting with acute febrile illness. During the initial COVID-19 vaccine trials, the vaccine was not tested in pregnant and lactating women, as clinical trials are not conducted in these groups. However, according to the recommendations of the American College of Obstetricians and Gynecologists, vaccination against COVID-19 should be offered to lactating women as well as to other persons belonging to the priority vaccination groups defined by ACIP [2]. According to these recommendations, women who have received the COVID-19 vaccine do not need to wean or avoid breastfeeding.

At the beginning of the vaccination program, there were no data on the safety of the COVID-19 vaccine in lactating women, its effects on the breastfed infant, or milk production and excretion. However, specialists recommended the mRNA vaccine against COVID-19 for breastfeeding mothers as safe both for the women and the breastfed babies. The mRNA vaccine contains only the genetic information necessary for the synthesis of the SARS-CoV-2 virus spike protein (S); it does not contain a weakened (attenuated) virus or virus that could replicate. Hence, the risk of adverse events in a child breastfed by a vaccinated mother are considered negligible.

Previous studies have shown the presence of antibodies in serum and breast milk after vaccination against tetanus, diphtheria, and pertussis [3,4], and after vaccination against influenza, with live, attenuated, and inactivated vaccines [5]. It is known that lactating mothers who receive these vaccines can transfer protective antibodies to their breastfed infants, so the relationship between vaccination against COVID-19 and antibodies in a mother’s serum and breast milk is an interesting subject for studies.

The US Food and Drug Administration (FDA) and the ACIP recommend leaving the decision on vaccination against COVID-19 to the women. The aim of our study was to determine the immune response to vaccination against COVID-19 in breastfeeding women and to evaluate the possible benefits for both mother and child.

## 2. Materials and Methods

### 2.1. Study Population

Information about the study was announced through peer contacts to breastfeeding women working in the health sector and published on social media. The study population included 32 breastfeeding women who, regardless of the study, had previously decided to be vaccinated due to their work in health care and the occupational risk of contracting COVID-19 infection. The control group included 28 breastfeeding women who were not vaccinated against COVID-19.

The study included a questionnaire to collect the mother’s and child’s ages at enrolment in the study, history of prior SARS-CoV-2 infection, the dates of COVID-19 vaccine doses, the occurrence of complications in the mother and child after each vaccine dose, and the type of breastfeeding (exclusively natural breastfeeding, mixed feeding—natural plus modified milk, breastfeeding during the expansion of the diet, or breastfeeding in a child with an extended diet).

### 2.2. Type of Vaccination

The study population was vaccinated with the mRNA vaccine BNT162b2 encoding the SARS-CoV-2 full-length spike (Pfizer–BioNTech), given intramuscularly in two doses 21 days apart in accordance with the local regulations and product characteristics. Each of the participants was qualified for SARS-CoV-2 vaccination, regardless of the study due to being healthcare professionals (HCP). The local vaccine allocation policy involved the early vaccination of HCPs with a BNT162b2 COVID-19 vaccine.

### 2.3. Ethics

The study was conducted in accordance with the Declaration of Helsinki ethical principles for medical research involving humans, and was approved by the Commission of Bioethics at Wroclaw Medical University (Poland)—agreement No KB 59/2021. All subjects gave written informed consent before study procedures were performed.

### 2.4. Sample Collection

Maternal serum samples (5 mL) and breast milk expressed on the day of serum collection (10 mL), after feeding the baby, were collected on day 8 ± 1 after the first dose of the vaccine, on day 22 ± 2 after the first dose of the vaccine (immediately prior to the second dose), on day 29 ± 3 after the first dose of the vaccine (which was also day 7 ± 3 after the second dose), and on day 43 ± 4 after the first dose of the vaccine (which was also day 21 ± 4 after the second dose). The samples of human milk were collected from the breast by manual expression and/or a breast pump after nursing (hindmilk) by complete breast emptying, once on the day of testing, at the same time (8:00–14:00). Serum samples were allowed to clot at room temperature for 15 min and were then centrifuged at 1000× *g* for 15 min. The aliquots of serum and milk samples were stored at −80 °C until analysis.

### 2.5. Analysis of Specific IgG, IgA, and IgM Antibodies

The immune response was assessed by determining the presence of anti-SARS-CoV-2 IgG and IgA antibodies in the maternal serum and breast milk. The concentrations of serum and breast milk anti-SARS-CoV-2 IgG were measured using quantitative enzyme-linked immunosorbent assay (anti-SARS-CoV-2 QuantiVac ELISA, EUROIMMUN). Serum samples were diluted 101 times, as suggested by the manufacturer, while breast milk samples were diluted 11 times. In the case of the detection of IgG >384 BAU/mL in a serum, a sample was further analyzed diluted 2091 times. The detection of IgG antibodies against SARS-CoV-2 is based on the S1 domain of the spike protein, including the immunologically relevant receptor-binding domain (RBD) as an antigen. RBD represents important target antigens for virus-neutralizing antibodies.

The test results of the measurement were converted into standardized binding antibody units (BAU/mL), which are in agreement with the First WHO International Standard for anti-SARS-CoV-2 immunoglobulin (NIBSC 20/136). The manufacturer recommends the following interpretation of the results for IgG in serum: <25.6 BAU/mL negative, ≥25.6 BAU/mL and <35.2 BAU/mL borderline, and >35.2 BAU/mL positive.

The presence of serum and breast milk anti-SARS-CoV-2 IgA and IgM was measured using semi-quantitative ELISA (EUROIMMUN). Serum samples were diluted 101 times, as suggested by the manufacturer, while breast milk samples were diluted 11 times. High serum IgA samples were additionally tested diluted 546 times. According to manufacturer guidelines, the results are presented as a ratio to the cut-off value, with the following interpretation of the results for the serum samples: <0.8 negative, ≥0.8 and <1.1 borderline, and >1.1 positive. The S1 domain of the spike protein is the antigen used in the ELISA for the detection of IgA. The ELISA for the detection of IgM antibodies against SARS-CoV-2 is based on the modified nucleocapsid protein (NCP).

The measurements were performed on the coded samples by researchers blinded to the information of vaccination status.

### 2.6. Statistical Analysis

Descriptive statistics were calculated for all demographics, clinical characteristics, and quantitative traits. All presented comparisons and correlations included non-normally distributed variables. Intergroup comparisons of continuous data were assessed using the non-parametric Mann–Whitney U test. Friedman’s rank test was used for comparing repeated measures of antibodies at multiple time points, followed by a post-hoc test according to Conover [6]. The correlations were performed using rank correlation (Spearman). A receiver operating characteristic (ROC) curve was used to define the cut-off value for breast milk IgG and IgA levels related to respective antibody serum positivity. The statistical test results, for which the *p*-values were less than 0.05, were considered significant. Statistical analysis was performed using Statistica (version 13.0 StatSoft) and MedCalc (version 20, MedCalc Software) software.

## 3. Results

### 3.1. Characteristics of the Study Population

The study samples were obtained from 60 enrolled participants between 7 January 2021 and 6 April 2021 (32 breastfeeding women vaccinated against COVID-19 and 28 breastfeeding women unvaccinated against COVID-19). The study population demographics and clinical characteristics, as well as adverse events following immunization (AEFI), are presented in Table 1. There were no differences between the study and control groups in the mothers’ (*p* = 0.08) and children’s ages (*p* = 0.77).

Moderate AEFI were observed fairly often in the women, especially after the second dose of the vaccine. There were no serious AEFI. Adverse events following immunization were observed rarely in children, and there is no proof that these symptoms were due to the vaccine. One mother reported a behavior change and increased tearfulness in a child after the first dose, and another mother reported sleeplessness of a child after the second dose.

### 3.2. Detection of Specific Anti-SARS-CoV-2 IgG and IgA Antibodies in Mother’s Serum and Breast Milk

Subjects with a history of prior SARS-CoV-2 infection based on the provided data and/or positive baseline levels of serum IgG antibodies were excluded from further analysis. These included four women from the vaccinated group (baseline serum IgG >384 BAU/mL, breast milk IgG 2.65 ± 2.71 BAU/mL, serum IgA >10.0, breast milk IgA 8.47 ± 2.53) and 12 women from the unvaccinated group (baseline serum IgG 96.52 ± 64.36 BAU/mL, breast milk IgG 1.18 ± 2.99 BAU/mL, serum IgA 1.68 ± 2.69, breast milk IgA 2.53 ± 2.46).

According to manufacturer recommendations, the serum samples were considered positive for IgG when the anti-SARS-CoV-2 IgG concentration was >35.2 BAU/mL. The serum samples were considered positive for IgA when the ratio was >1.1.

**Figure 1:**
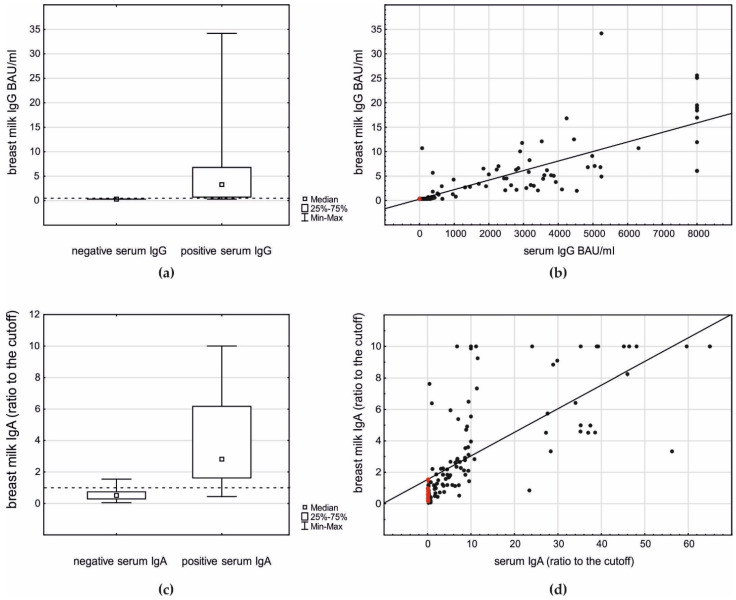
The relationship between breast milk IgG and IgA antibodies and their serum levels in postvaccination samples: (**a**) breast milk IgG level related to the antibody presence in serum (Mann–Whitney U test *p* < 0.001); (**b**) correlation between serum and breast milk IgG (r_S_ 0.89, *p* < 0.001): red dots—control group; black dots—study group; (**c**) breast milk IgA level related to the antibody presence in serum (Mann–Whitney U test *p* < 0.001); and (**d**) correlation between serum and breast milk IgA (r_S_ 0.83, *p* < 0.001): red dots—control group; black dots—study group.

Breast milk IgG and IgA antibodies were observed in postvaccination samples, and their occurrence was highly related to their presence in the serum (Figure 1a,c).

When serum IgG-negative women were considered, none of their breast milk samples presented a detectable level of IgG. In the case of 68 (74%) of the breast milk samples of serum IgG-positive women, the IgG level was detectable (6.50 ± 6.74, median 4.7, and maximum 34.2 BAU/mL, *p* < 0.001; Figure 1a) and highly correlated to the serum IgG level (r_S_ 0.89, *p* < 0.001; Figure 1b). The ROC analysis revealed that the detection of breast milk IgG >0.4 BAU/mL is strongly related to the respective serum IgG positivity (AUC 0.92, *p* < 0.001).

The breast milk ratio of IgA to the cut-off value was higher in the serum IgA-positive women (4.18 ± 3.26, median 2.8, and maximum >10) than in the serum IgA-negative women (0.56 ± 0.37, median 0.5, and maximum 1.6, *p* < 0.001, Figure 1c). The ROC analysis revealed that the detection of breast milk IgA with a ratio >1.0 is related to serum IgA positivity (AUC 0.80, *p* < 0.001). Moreover, in the case of IgA, a strong correlation between the serum and breast milk level was observed (r_S_ 0.83, *p* < 0.001; Figure 1d).

None of the serum samples (including those taken postvaccination) was positive for anti-SARS-CoV-2 (NCP) IgM antibodies (0.11 ± 0.06, median 0.09, and maximum 0.32), supporting the hypothesis that the observed postvaccination formation of IgG and IgA antibodies was due to the vaccine, not to accidental postvaccination SARS-CoV-2 infection. Accordingly, none of the breast milk samples studied presented a detectable level of anti-SARS-CoV-2 (NCP) IgM antibodies (0.02 ± 0.02, median 0.02, and maximum 0.06).

### 3.3. Analysis of Specific Anti-SARS-CoV-2 IgG and IgA Antibodies Induced by a Vaccination

After the exclusion of previously SARS-CoV-2 infected subjects, all baseline serum and breast milk samples were anti-SARS-CoV-2 IgG and IgA antibody negative, with no differences in the absolute values observed in the study group on day 8 ± 1 and in the control group (Table 2).

**Figure 2:**
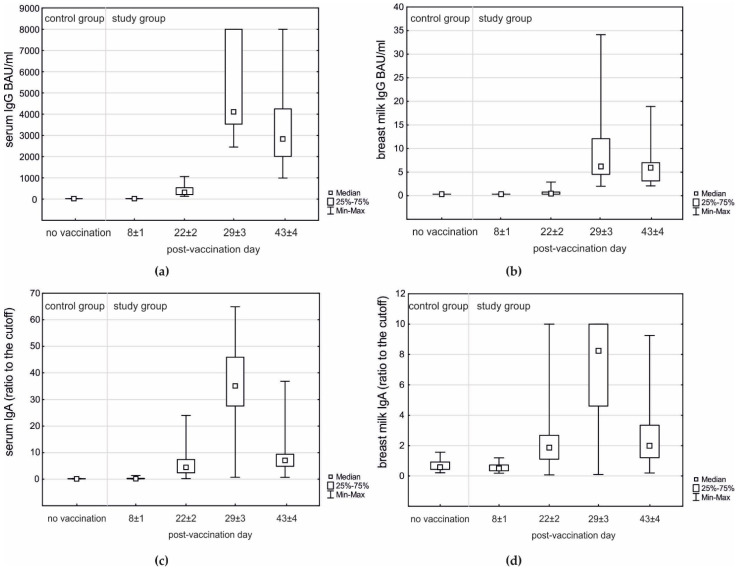
Postvaccination IgG and IgA antibodies in serum and breast milk samples of the study group (*n* = 28) compared to unvaccinated control group (*n* = 16): (**a**) Serum IgG—day 8 ± 1 (baseline) vs. no vaccination; Mann–Whitney *p* = 0.152; days 8 ± 1, 22 ± 2, 29 ± 3, and 43 ± 4; Friedman’s rank test *p* < 0.001 with post-hoc all pairwise comparisons *p* < 0.05. (**b**) Breast milk IgG—day 8 ± 1 (baseline) and no vaccination below detection limit; days 8 ± 1, 22 ± 2, 29 ± 3, and 43 ± 4; Friedman’s rank test *p* < 0.001 with post-hoc all pairwise comparisons *p* < 0.05. (**c**) Serum IgA—day 8 ± 1 (baseline) vs. no vaccination; Mann–Whitney *p* = 0.129; days 8 ± 1, 22 ± 2, 29 ± 3, and 43 ± 4; Friedman’s rank test *p* < 0.001 with post-hoc all pairwise comparisons *p* < 0.05. (**d**) Breast milk IgA—day 8 ± 1 (baseline) vs. no vaccination; Mann–Whitney *p* = 0.371; days 8 ± 1, 22 ± 2, 29 ± 3, and 43 ± 4; Friedman’s rank test *p* < 0.001 with post-hoc pairwise comparisons *p* < 0.05, except for no difference between days 22 ± 2 and 43 ± 4.

Positive serum IgG was observed in all vaccinated women on day 22 ± 2; however, positive breast milk IgG samples were observed in 14/28 (50%) on day 22 ± 2 and in all women on days 29 ± 3 and 43 ± 4. The highest concentrations of serum IgG, serum IgA, breast milk IgG, and breast milk IgA were observed on day 29 ± 3 with a decrease on day 43 ± 4 (Table 3; Figure 2).

The type of breastfeeding did not have an influence on the level of IgG (*p* = 0.428) or IgA (*p* = 0.522) in breast milk. The presence and severity of AEFI in mothers after the first or second dose of the vaccine also did not influence the breast milk level of IgG (*p* = 0.782 and *p* = 0.342, respectively) or IgA (*p* = 0.435 and *p* = 0.431, respectively).

## 4. Discussion

In our study, both the IgG and IgA antibodies increased in the serum and breast milk after vaccination, with the highest concentrations of all antibodies on day 29 ± 3 with a decrease on day 43 ± 4. These observations are consistent with another study that was recently published. This study found the robust secretion of SARS-CoV-2-specific IgA and IgG antibodies in breast milk for 6 weeks after vaccination [7]. Moreover, antibodies against SARS-CoV-2 found previously in the breast milk of women infected with COVID-19 showed strong neutralizing effects, suggesting a potential protective effect against infection in the infants [8].

Therefore, our study provided the basis for a better understanding of the immune response to a COVID-19 vaccination in breastfeeding women and its possible effects on the breastfed child. The protection provided to the child of a vaccinated mother was not studied, but it is well known that lactating mothers, who receive a vaccination against tetanus, diphtheria, and pertussis [3,4], or against influenza [5], can transfer protective antibodies to their nursing infant.

One of these studies demonstrated that vaccine-generated antibodies were present in all umbilical cord blood and breast milk samples, with higher titers than those induced by SARS-CoV-2 infection during pregnancy [9]. Therefore, vaccine-induced immune responses were significantly greater than the response to natural infection. SARS-CoV-2-specific IgG (but not IgA) antibodies increased in maternal blood and breast milk with a vaccine boost [9].

The immune properties of the mother are also transferred to the breastfed infant in the form of IgG and secretory IgA (sIgA) in maternal milk [10]. Breast milk attains the highest concentration of IgG antibodies in the colostrum, and their concentration drops after the first month of life and stops abruptly with weaning [11]. The vaccination of mothers causes antibodies to be present in the milk. The immunization of mothers against Respiratory Syncytial Virus (RSV) triggered the presence of IgG antibodies in their breast milk, providing protection to the infant against the main cause of respiratory infection during the first year of life [12]. IgA antibodies coat the gastrointestinal tract and respiratory mucosa, and block the entrance of foreign antigens and viruses. In respiratory tract infections caused by RSV, protection is mediated by polymeric IgA antibodies to a protein of the RSV surface membrane, inhibiting virus replication [13]. sIgA is the most abundant antibody in breast milk that provides adequate specific protection against pathogens, including viruses. The specificity of sIgA depends on the maternal immune response to previous infection, which probably explains the low infection rate or milder symptoms seen in infected infants breastfed by mothers infected with SARS-CoV-2 [14]. A similar mechanism can be involved in the immune response after maternal vaccination [15]. sIgA is also influenced by other maternal factors, including nutrition, genetics, severity of infection, and the elapse of time between the infection or vaccination and the sample collection [16].

It is noteworthy that high levels of immunoglobulins were observed in the day 8 ± 1 serum and breast milk samples from vaccinated women previously infected with SARS-CoV-2. However, the limited number of vaccinated recovered subjects in our study did not allow us to perform any reliable analysis of the impact of vaccination in this group of subjects.

As there were no serious side effects in the children after the mothers’ vaccinations, and the presence of IgG and IgA antibodies in the breast milk was shown, the study gives further evidence on the importance of vaccination against COVID-19 in breastfeeding women. Therefore, the study may affect future recommendations for vaccination against COVID-19 in the general population of breastfeeding mothers.

### Limitation of the Study

It should be noted, nevertheless, that our study has some limitations. The study group was small; however, based on the results, it was possible to draw conclusions.

The study showed increased levels of specific IgG and IgA antibodies in serum and breast milk; however, the protection they provide to the child of the vaccinated mother was not studied. Lactating mothers, who receive a vaccination against SARS-CoV-2 and regularly breastfeed their children, may transfer protective antibodies to their nursing infants, who acquire passive immunity.

As this study did not evaluate the neutralization capacity of IgG and IgA, future investigation is needed to evaluate whether lactating mothers who have had a COVID-19 vaccine transfer protective antibodies to their nursing infants.

IgM can be produced during SARS-CoV-2 infection or after a vaccination in either serum or breast milk [9,17,18]. However, the assay we used for the detection of IgM antibodies against SARS-CoV-2 is based on the modified nucleocapsid protein (NCP). The test was not expected to detect IgM antibodies resulting from a response to vaccination with a SARS-CoV-2 spike sequence, but rather to serve as a measure of an immune response to potential infection with SARS-CoV-2 during the first weeks after vaccination.

## 5. Conclusions

Based on our study results and conducted statistical analysis, the following conclusions were drawn:

1. The immune response to the vaccination against SARS-CoV-2 is strongest 7 ± 3 days after the second dose (29 ± 3 days after the first dose of the vaccine).
2. The levels of IgA and IgG antibodies specific to the SARS-CoV-2 spike antigen in breast milk and serum samples from mothers after a COVID-19 vaccine were positively correlated.

## Figures and Tables

**Table 1:** Study population characteristics.

Variables	Vaccinated (*n* = 32)	Unvaccinated (*n* = 28)
Mother’s age (years)	33.3 ± 2.9 (33.6)	31.7 ± 3.3 (31.5)
Child’s age at study enrolment (months)	8.8 ± 6.8 (6.4)	10.1 ± 8.7 (7.8)
Type of breastfeeding		
1 Exclusively natural breastfeeding	14 (43.8%)	13 (46.4%)
(exclusively natural breastfeeding at the time of 1st vaccine dose, breastfeeding during the expansion of the diet at the time of 2nd vaccine dose)	2 (6.3%)	―
2 Mixed feeding—natural plus modified milk	2 (6.3%)	1 (3.6%)
3 Breastfeeding during the expansion of the diet	7 (21.8%)	4 (14.3%)
4 Breastfeeding in a child with an extended diet	9 (28.1%)	10 (35.7%)
Prior SARS-CoV-2 infection	4 (12.5%)	12 (42.9%)
Time between infection and study enrolment (days)	94 ± 1.73 (95.0)	104.4 ± 43.8 (108.5)
Infection without symptoms	1 (3.1%)	4 (14.3%)
Symptoms		
Fever	0	3 (10.7%)
Fry cough	0	0
Dyspnea	0	1 (3.6%)
Fatigue	2 (6.2%)	7 (25%)
Headache	1 (3.1%)	5 (17.9%)
Muscle aches	2 (6.2%)	5 (17.9%)
Arthralgia	2 (6.2%)	5 (17.9%)
Chills	0	1 (3.6%)
Loss of taste or smell	2 (6.2%)	6 (21.4%)
Skin rash or discoloration of the fingers and toes	0	0
Feeling unwell	2 (6.2%)	5 (17.9%)
Sore throat	2 (6.2%)	1 (3.6%)
AEFI in the mother after each vaccine dose: 1st *n*(%)/2nd *n*(%)	25 (78.1%)/28 (87.5%)	―
Pain at the injection site	24 (75%)/25 (78.1%)	―
Fatigue	9 (28.1%)/17 (53.1%)	―
Headache	10 (31.3%)/18 (56.3%)	―
Muscle aches	6 (18.8%)/19 (59.4%)	―
Arthralgia	5 (15.6%)/10 (31.3%)	―
Chills	3 (9.4%)/14 (43.8%)	―
Fever	2 (6.3%)/8 (25%)	―
Swelling at the injection site	4 (12.5%)/6 (18.8%)	―
Redness at the injection site	5 (15.6%)/4 (12.5%)	―
Nausea	1 (3.1%)/4 (12.5%)	―
Enlarged lymph nodes	0/6 (18.8%)	―
Feeling unwell	6 (18.8%)/18 (56.3%)	―
AEFI in child after each vaccine dose: 1st *n*(%)/2nd *n*(%)	1 (3.1%)/1 (3.1%)	―
Low-grade fever	0/0	―
Fever	0/0	―
Behavior change	1 (3.1%)/0	―
Increased tearfulness	1 (3.1%)/0	―
Increased muscle tone	0/0	―
Vomiting	0/0	―
Diarrhea	0/0	―
Other (sleeplessness)	0/1 (3.1%)	―

Data are presented as *n* (%) or mean ± SD (median).

**Table 2:** Baseline serum and breast milk IgG and IgA antibodies in study and control groups.

Antibodies	Study Group (No Prior Infection) (*n* = 28)	Control Group (No Prior Infection) (*n* = 16)	*p* Value *
Serum IgG (BAU/mL)	4.62 ± 3.57 (3.2, 3.2–4.1)	3.41 ± 0.80 (3.2, 3.2–3.2)	0.152
Breast milk IgG (BAU/mL)	n.d.	n.d.	-
Serum IgA (ratio)	0.33 ± 0.37 (0.19, 0.14–0.33)	0.17 ± 0.07 (0.16, 0.12–0.22)	0.129
Breast milk IgA (ratio)	0.55 ± 0.32 (0.49, 0.32–0.71)	0.69 ± 0.39 (0.57, 0.42–0.90)	0.371

Data are presented as mean ± SD (median, IQR); n.d.—not detected; IgG breast milk lower detection limit was 0.32 BAU/mL; * study vs. control group (no prior infection); Mann–Whitney U test.

**Table 3:** Postvaccination serum and breast milk IgG and IgA antibodies.

Antibodies	Day 8 ± 1 (Baseline)	Day 22 ± 2	Day 29 ± 3	Day 43 ± 4	*p* Value *
Serum IgG (BAU/mL)	4.62 ± 3.57 (3.2, 3.2–4.1)	373.6 ± 237.7 (302, 197–520)	5055.8 ± 2057.6 (4108, 3524–8000)	3276.5 ± 1803.3 (2822, 1997–4241)	<0.001
Breast milk IgG (BAU/mL)	n.d.	0.66 ± 0.57 (0.42, 0.32–0.76)	10.13 ± 8.55 (6.2, 4.5–12.1)	6.43 ± 4.33 (5.9, 3.1–7.0)	<0.001
Serum IgA (ratio)	0.33 ± 0.37 (0.19, 0.14–0.33)	5.55 ± 4.59 (4.53, 2.44–7.42)	35.16 ± 17.52 (35.3, 27.7–46.0)	9.37 ± 8.08 (7.1, 4.9–9.4)	<0.001
Breast milk IgA (ratio)	0.55 ± 0.32 (0.49, 0.32–0.71)	2.50 ± 2.42 (1.9, 1.1–2.7)	7.14 ± 3.04 (8.2, 4.6–10.0)	2.73 ± 2.13 (2.0, 1.2–3.3)	<0.001

Data are presented as mean ± SD (median, IQR); study group: *n* = 28; n.d.—not detected; IgG breast milk lower detection limit was 0.32 BAU/mL; * Friedman’s rank test.

## Data Availability

Not applicable.
